# Next-Generation Sequencing Coupled With *in situ* Hybridization: A Novel Diagnostic Platform to Investigate Swine Emerging Pathogens and New Variants of Endemic Viruses

**DOI:** 10.3389/fvets.2019.00403

**Published:** 2019-11-15

**Authors:** Talita P. Resende, Lacey Marshall Lund, Stephanie Rossow, Fabio A. Vannucci

**Affiliations:** ^1^Department of Veterinary and Biomedical Sciences, College of Veterinary Medicine, University of Minnesota, St. Paul, MN, United States; ^2^Veterinary Diagnostic Laboratory, College of Veterinary Medicine, University of Minnesota, St. Paul, MN, United States

**Keywords:** pig, diagnosis, emerging infectious diseases, NGS-ISH, PCV3, PPV2, SVA

## Abstract

Next generation sequencing (NGS) can be applied to identify and characterize the entire set of microbes within a sample. However, this platform does not provide a morphological context or specific association between the viral or bacterial sequences detected and the histological lesions. This limitation has generated uncertainty whether the sequences identified by NGS are actually contributing or not for the clinical outcome. Although *in situ* hybridization (ISH) and immunohistochemistry (IHC) can be used to detect pathogens in tissue samples, only ISH has the advantage of being rapidly developed in a context of an emerging disease, especially because it does not require development of specific primary antibodies against the target pathogen. Based on the sequence information provided by NGS, ISH is able to check the presence of a certain pathogen within histological lesions, by targeting its specific messenger RNA, helping to build the relationship between the pathogen and the clinical outcome. In this mini review we have compiled results of the application of NGS-ISH to the investigation of challenging diagnostic cases or emerging pathogens in pigs, that resulted in the detection of porcine circovirus type 3, porcine parvovirus type 2, Senecavirus A, and *Mycoplasma hyorhinis*.

## Introduction

The U.S. swine industry has evolved from small independent farming operations to integrated large systems (1). Along with this process, the production systems have faced emerging health challenges, particularly regarding virus diseases (2). In addition, virus-associated syndromes and disease complexes have become more common due to the involvement of multiple pathogens or virus subtypes in the same tissue (2). The early identification of pathogens in pig herds is crucial for the decision-making process in regards of disease control, prevention, strategy of treatment and, therefore, mitigation of the impact of a particular disease (3). Next-generation sequencing has been recently used to detect nucleotide sequences in challenging diagnostic cases, but it does not provide morphological context that would allow the association of a specific viral sequences with the histological lesions. This scenario generates uncertainty whether the sequences identified by NGS are actually contributing for the clinical signs. While immunohistochemistry may overcome this limitation by detecting antigens in association with histological lesions, it requires specific antibodies that are not always promptly available commercially (4). Alternatively, *in situ* hybridization assay allows the detection of nucleotide sequences in histological sections without requiring the development of such antibodies.

Traditional diagnostic methods are still extremely important to veterinary diagnostics medicine. However, the turnaround time for results that are crucial during diagnostic investigations of unsolved cases can be much to slow. Q-PCR is an assay with a short turnaround that offers an indirect quantification of the amount of a microorganism in the sample, but it is not able to distinguish whether a given pathogen was viable in the sample, whether its presence is associated with histological lesions of if the pathogen/lesions association correlates with the clinical signs. Bacterial and viral isolation is considered the gold standard method for the definitive diagnostics of numerous infectious diseases, but these classic approaches can be time consuming. Also, isolation of an emerging pathogen faces obstacles, such as what is the susceptible cell line or media for isolation, if it is a caused by a non-culturable agent and are there multiple agents involved in the disease syndrome (5).

Tissue-based diagnosis is undeniably one of the most important approaches for the identification of a potential role of the pathogen as the etiologic agent of that disease. The histological lesions recognized by the pathologist can be associated with the presence of a given pathogen detected by immunohistochemistry within the lesions (4). Nevertheless, when dealing with an emerging disease, production of a sensible and specific antibody and the optimization of the immunohistochemistry protocol can take several months, delaying effective actions to control a particular emerging disease. This mini-review focuses on the development and application of a new platform of diagnostics that combine next-generation sequencing and *in situ* hybridization for investigating unsolved diagnostic cases in swine.

## Next-Generation Sequencing and *in situ* Hybridization

Sequence technologies have risen in the past few years as a meaningful ancillary tool for diagnostic investigation of infectious diseases. Next generation sequencing (NGS) is one of the most recent sequencing approaches included in the range of diagnostic methods for veterinary diagnostic investigations (6–8). The NGS advantage in relation to simple sequencing relies on a *de novo* or mapping assembly of sequenced regions, dismissing the use of specific targets to build a complete genome. With NGS, genome fragments of virtually all the microorganisms present in the clinical samples are sequenced and then, re-assembled based on genome data bases publicly available. These characteristics of NGS make it very convenient for investigation of infectious disease outbreaks in which the etiologic agent is unknown. Although extremely sensitive, NGS lacks the association of the presence of a microorganism and the type of histological lesion. Since the development of specific antibodies for immunohistochemistry requires a long period of time, there was a demand for a diagnostic method that would allow the association of the genomes detected by NGS with a tissue lesion.

**Figure 1:**
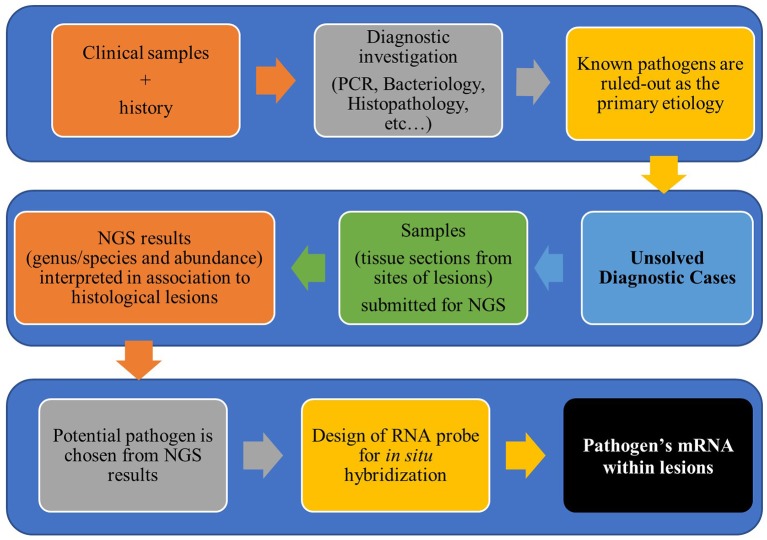
Workflow for diagnostic investigation of unsolved cases using next-generation sequencing and *in situ* hybridization.

The usefulness of *in situ* hybridization (ISH) to detect pathogens within histological lesions in pigs has been recognized since the 1990's (9–12). At first, ISH probes were radioactively labeled, but due to the risk of manipulation and low sensitivity of the test, alternative labeling systems were developed (4), such as digoxigenin, biotin, dinitrophenol, and fluorescence (also named as FISH). Although the traditional ISH assays have satisfying specificity, their sensitivity has also been a target of discussion even with non-radioactively labeled probes, especially when the target was a short nucleotide sequence (4, 10, 13, 14). Recently, a new approach for increased ISH sensitivity has been developed (15). This improved method is based on a signal amplification system that allows visualization of a single molecule in paraffin embedded sections. Since then, various publications have proven the applicability of this new ISH method for research studies in veterinary medicine (16–21). In those cases, the design of ISH probes was based on the nucleotide sequences of endemic or well-described microorganisms. Withal, in cases in which the involvement of known pathogens has been ruled out by other methods, there is a need of an auxiliary method for the detection of possible new emergent pathogens or variant of known pathogens in clinical samples. Therefore, the association of NGS and ISH has been extremely advantageous to overcome those limitations (Figure 1).

## Application

### Vesicular Lesions Caused by Senecavirus A

The years of 2014 and 2015 were marked in the swine industry of the USA, Canada, China, and Brazil by outbreaks of diseases characterized by vesicular lesions on the coronary band and snouts of sows and growing pigs and acute neonatal pig deaths (22–25). In addition to the impact of the disease in pig herds itself, the vesicular lesions are clinically indistinguishable from foot-and-mouth disease (FMD) and other vesicular diseases.

Diagnostic investigations ruled out the most common vesicular diseases of pigs (swine vesicular disease, vesicular stomatitis and vesicular exanthema), as well as FMD. Then, samples were submitted for NGS. NGS revealed genome sequences with high similarity to Senecavirus A (SVA) available in GenBank (22). Since then, SVA has been confirmed as the etiologic agent of those outbreaks of vesicular diseases in pig herds in North America and South America (26, 27), and a commercial kit for SVA qRT-PCR was developed.

Although SVA has been associated with swine idiopathic vesicular disease in Canada and the USA since 2008, anti-SVA antibodies were not commercially available for IHC for investigation of SVA outbreaks in 2014. Based on the genome sequences identified by NGS, ISH probes were designed to investigate the presence of SVA within the histological lesions from RT-qPCR positive samples, including tissue samples from piglets affected by acute neonatal losses (17). Lesions in the skin of sows with snout and coronary band vesicles consistently associated with low Ct values for SVA in the RT-qPCR and a strong positive label in ISH (17). Samples from piglets affected by the acute neonatal losses did not showed histological lesions, with exception of erosive lesions of tongues from piglets (17, 23), which were also ISH positive for SVA-mRNA.

ISH has also been valuable for investigation of the pathogenesis of SVA infection in pigs. Vesicular lesions were reproduced by the experimental infection of 15-weeks-old pigs with a contemporary SVA isolate obtained from a lesion swab of a finishing pig with vesicular disease (18). Histological lesions were observed in skin (vesicles) and associated with ISH positive signals as previously reported. All other tissues were histologically normal, except lymphoid tissues, in which lymphoid hyperplasia was observed. ISH positive signals for SVA were observed in tonsils, which had higher amounts of nucleic acids determined by RT-qPCR (18).

### PCV2- and PCV3-Associated Diseases

Pigs from many countries have been suffering from clinical syndromes caused by porcine circovirus type 2 (PCV2). PCV2 associated disease was first described in the early 1990's (28). The clinical signs of PCV2 are associated with the well-recognized manifestations of the infection [post-weaning multisystemic wasting syndrome (PMWS), Porcine dermatitis and nephropathy syndrome (PDNS), reproductive disease, enteric disease, lung disease] although subclinical infections are also common (29). Since the development of a vaccine against PCV2, the clinical signs of PCV2-associated diseases has been diminished (29). It was in 2016 when the new porcine circovirus was found in pigs exhibiting clinical signs similar to the PCV2-associated diseases.

Cases of post-weaned pigs with unspecific clinical signs, mainly characterized by weight loss, failure to thrive and occasionally respiratory distress were presented at the Veterinary Diagnostic Laboratory at the University of Minnesota (UMN-VDL). Although the majority of the clinical signs and histological lesions were attributed to the presence of known pathogens identified by traditional diagnostic tests, lymphoplasmacytic and histiocytic myocarditis, vasculitis and interstitial pneumonia observed in affected pigs were still lacking an etiologic explanation. Tissue samples were submitted to NGS and a “porcine circovirus-like” sequence was consistently identified. From the sequences identified by NGS, a sequence of 200 bases with high similarity to the genus *Circovirus*, was used to design a ISH probe, in order to confirm the presence of the proposed virus within histological lesions. PCV3 mRNA demonstrated by hybridization signals was observed in cardiomyocytes and in wall of arteries with inflammation (30). PCV3 has then been recognized as a new *Circovirus* species by the International Committee on Taxonomy of Viruses (ICTV) (31).

Since then, primers for q-PCR were developed based on the PCV3 genome sequence and have been used in countries from Asia (32–34), Europe (35, 36), and South America (37, 38). PCR positive results and ISH positive signals were detected in tissues sections from sows with PDNS and reproductive failure, in tissues from aborted fetuses, and in diverse samples from pigs with PMWS, especially in myocardium and arteries (39, 40). Although viral isolation is still lacking, PCV3 has been proposed as the etiologic agent of clinical syndrome associated with the histological lesions described above, based on the molecular guidelines for microbiological etiologic causation as suggested by Fredricks and Relman (41).

Although PCV2 could be ruled out as a potential etiologic agent in cases of PMWS, PNDS, and reproductive failure, there was still a possibility of a co-infection of PCV2 and PCV3, especially due to the endemic distribution of both viruses within the pig population. Hence, a duplex-ISH was developed to allow the simultaneous detection of both viruses in pig samples (37). From a total of 477 tissue samples recovered from the UMN-VDL historical cases, 9% (*n* = 43) were positive for both viruses, PCV2 and PCV3 by ISH (37). Both viruses were predominantly observed in germinal centers in lymph nodes, in peritarteriolar lymphoid tissue in the spleen, in lymphohistiocytic infiltrates of heart arterioles and also in peri-bronchiolar lymphoid cuffs (37). However, it was noted that lymphoid depletion is not a characteristic of PCV3 infection, as it is for PCV2. These results highlight the challenges for interpreting PCR results when animals are positive for PCV2 and PCV3 and reinforce the need of qualified pathologists to interpret the histological lesions and the possible association with the agent identified by NGS.

### *Mycoplasma hyorhinis*-Associated Conjunctivitis

Recent outbreaks of swine conjunctivitis have been reported to the University of Minnesota Veterinary Diagnostic lab. After ruling out the most common causes of infectious conjunctivitis in pigs (pseudorabies, swine influenza, porcine cytomegalovirus, and Chlamydia) and discarding the possibility of a non-infectious cause palpebral conjunctiva from affected pigs were submitted for NGS. NGS results indicated a high proportion of *M. hyohrinis* genome in the samples, which were confirmed by qPCR. In order to verify whether *M. hyorhinis* was present within the lesions, and due to a lack of antibodies anti-*M. hyorhinis* for immunohistochemistry, ISH probes were designed based on the 16S sequence of *M. hyorhinis*. Hybridization signals were observed in samples from affected pigs, but not in samples from non-affected animals from unrelated non-affected herds (42). These results corroborate past investigations that indicated *M. hyorhinis* as the etiologic agent of swine conjunctivitis (43, 44).

### Porcine Parvovirus Type 2-associated With Perivasculitis

A novel porcine parvovirus, parvovirus type 2 (PPV2) was originally identified in Myanmar in 2001 in a serum sample (45), and since then, PPV2 has been detected in various pig samples (46–48). There has been reports of a positive correlation of PPV2 detection and poor performance in affected pigs (47) and of presence of PPV2 in lung tissues from pigs with respiratory clinical signs and PCR positive for PCV2. PPV2 was identified by direct in *in situ* PCR in pulmonary lesions described as vascular thickness caused by lymphocytic infiltration, reduced alveolar spaces and epithelial damage, without a direct correlation with PCV2 detection in the same tissues (49). Cases of poor growth performance in nursery pigs associated with systemic perivascular inflammation were studied for potential causative agent at the UMN-VDL. Due to the lack of detection of known pathogens in samples from the affected pigs, tissue samples were submitted for NGS. The high proportion of PPV2 sequences identified by NGS along with compatible histological findings suggested the involvement of PPV2 in the cases. Samples were then tested by RT-qPCR and positive tissues were submitted to ISH. PPV2 ORF mRNA was chosen as the target for ISH probes. ISH PPV2 signals were observed in an association with the histological lesions in various tissues (lung, joint and subcutaneous tissues) within the cytoplasm of endothelial cells and in lymphoid follicles of the lymph nodes and broncho-associated lymphoid tissues (50). These results represent an important advancement for understanding the potential role of PPV2 in emerging systemic syndromes in nursery and finishing pigs.

### Porcine Sapelovirus in Pigs With Polioencephlomielitis

Outbreaks of atypical neurological disease were reported in swine herds of the United States in the past few years. Clinical signs of anorexia, compromised movement, decreased responso se stimuli and mental dullness were associated with severe lymphoplasmacytic and necrotizing polioencephalomyelitis with multifocal areas of gliosis and neuron satellitosis, suggestive of a neurotropic viral infection. Due to the lack of detection of the most commonly viruses associated to neurological diseases in pigs, such as pseudorabies virus and atypical porcine pestivirus, porcine reproductive and respiratory syndrome virus and porcine circovirus, samples of brainstem and spinal cords from affected pigs were used NGS. NGS results identified porcine sapelovirus and absence of other or novel pathogens. By ISH, Sapelovirus A mRNA was detected in neurons and nerve roots of the spinal cord of affected pigs (51).

## Final Considerations

The incidence of swine emerging, and reemerging diseases have increased in the past few years. Traditional ancillary tests are routinely used to investigate the involvement of known pathogens when samples from outbreaks are sent for diagnostic investigation. However, one of the characteristics of traditional ancillary tests is that they are designed to detect known pathogens. The association of NGS-ISH has been instrumental on the investigation of etiologic causes of cases in which the traditional ancillary tests did not identified involvement of known pathogens. The UMN-VDL, one of the most important diagnostic laboratories for swine infectious diseases in the US, is a pioneer in using NGS-ISH as a tool investigate possible emerging diseases. Nevertheless, as happens to new technologies are being implemented and optimized, NSG-ISH has its drawbacks. Although NGS is currently cheaper, faster and more assessible to non-research purposes, it is still a technology that requires structured laboratories, equipment and, most importantly, qualified professionals to interpret the relevance of the several microorganisms' sequences identified by the sequencing in the swine samples. ISH also has its own limitations. By targeting mRNA, ISH helps to determine whether the virus is metabolically active within lesions. However, the approach relies on targeting a mRNA that corresponds to the DNA sequence identified by the NGS. If the chosen DNA sequence is not been translated in mRNA, the absence of positive signals can mean a false negative. In addition, ISH method used in combination with NGS it is still relatively expensive due to dependency on a single manufacturer that detains the intellectual property of the ISH technique. Nevertheless, we anticipate that the in the next few years both the demand for best diagnostic approaches for emerging diseases will stimulate both methodologies to evolve in regards of their feasibility and costs, making the NGS-ISH an important tool for identifying pathogens in swine emerging diseases.

In conclusion, the NGS-ISH diagnostic platform presented here combines the comprehensive unbiased detection of nucleic acid sequences with the morphological context shown in the histological lesions. This characteristic has been specifically important to diagnose infectious diseases in which the clinical and laboratory findings are not able to specific determine the primary pathogen involved in the clinical outcome. Additionally, the ability to detect sequences from previously unknown agents through NGS allows the design and subsequent identification of emerging pathogens within tissue sections, by targeting mRNA using ISH assay. This rapid diagnostic response is critical to implement control measurements and mitigate economic losses in the swine industry.

## Author Contributions

TR conducted the literature review and wrote the manuscript. LM and SR helped revising the manuscript, adding important scientific content, and refining the interpretation of the results. FV conceived of the idea of the review and helped revising the manuscript to add important scientific content and refine the interpretation of the results. All the authors reviewed the final version of the manuscript and agreed to its submission.

### Conflict of Interest

The authors declare that the research was conducted in the absence of any commercial or financial relationships that could be construed as a potential conflict of interest.
